# QSAR Study, Molecular Docking and Molecular Dynamic Simulation of Aurora Kinase Inhibitors Derived from Imidazo[4,5-*b*]pyridine Derivatives

**DOI:** 10.3390/molecules29081772

**Published:** 2024-04-13

**Authors:** Yang-Yang Tian, Jian-Bo Tong, Yuan Liu, Yu Tian

**Affiliations:** 1 College of Petroleum Engineering, Xi’an Shiyou University, Xi’an 710065, China; tianyangyang2012@163.com; 2 Shaanxi Key Laboratory of Advanced Stimulation Technology for Oil & Gas Reservoirs, Xi’an 710065, China; 3 College of Chemistry and Chemical Engineering, Shaanxi University of Science and Technology, Xi’an 710021, China; 210812097@sust.edu.cn (Y.L.); tianyu@sust.edu.cn (Y.T.)

**Keywords:** QSAR, Aurora kinase, molecular designing, molecular docking, molecular dynamic simulation, free energy landscape, MM/PBSA, ADMET

## Abstract

Cancer is a serious threat to human life and social development and the use of scientific methods for cancer prevention and control is necessary. In this study, HQSAR, CoMFA, CoMSIA and TopomerCoMFA methods are used to establish models of 65 imidazo[4,5-*b*]pyridine derivatives to explore the quantitative structure-activity relationship between their anticancer activities and molecular conformations. The results show that the cross-validation coefficients *q*^2^ of HQSAR, CoMFA, CoMSIA and TopomerCoMFA are 0.892, 0.866, 0.877 and 0.905, respectively. The non-cross-validation coefficients *r*^2^ are 0.948, 0.983, 0.995 and 0.971, respectively. The externally validated complex correlation coefficients *r*^2^*_pred_* of external validation are 0.814, 0.829, 0.758 and 0.855, respectively. The PLS analysis verifies that the QSAR models have the highest prediction ability and stability. Based on these statistics, virtual screening based on *R* group is performed using the ZINC database by the Topomer search technology. Finally, 10 new compounds with higher activity are designed with the screened new fragments. In order to explore the binding modes and targets between ligands and protein receptors, these newly designed compounds are conjugated with macromolecular protein (PDB ID: 1MQ4) by molecular docking technology. Furthermore, to study the nature of the newly designed compound in dynamic states and the stability of the protein-ligand complex, molecular dynamics simulation is carried out for N3, N4, N5 and N7 docked with 1MQ4 protease structure for 50 ns. A free energy landscape is computed to search for the most stable conformation. These results prove the efficient and stability of the newly designed compounds. Finally, ADMET is used to predict the pharmacology and toxicity of the 10 designed drug molecules.

## 1. Introduction

Cell division is a biological marker, which is strictly regulated by a large number of proteins. The occurrence of cancer is due to the uncontrolled division, growth and spread of cancer cells. These cells go beyond their usual boundaries, then attack the adjacent parts of the body and spread to other organs. This can occur in any part of the body, leading to the death [1]. Using scientific methods to prevent and control cancer has become one of the most important public health targets in the world [2]. In the regulatory protein network of cell division, Aurora kinase plays an important role in cell division by controlling chromatin separation, which can lead to genetic instability, often accompanied by cancer [3].

**Figure 1:**
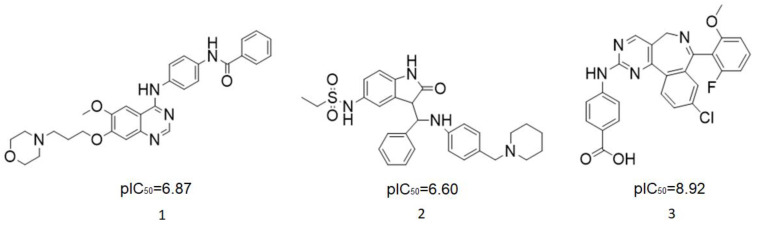
Several Aurora kinase inhibitors that have entered clinical trials.

Aurora kinase, a member of serine/threonine family, is responsible for cell cycle regulation. Aurora kinase is involved in many mitotic processes, such as centrosome maturation and separation, spindle formation and stability, chromosome separation and cytokinesis, so as to coordinate the orderly mitosis. Abnormal expression or function of Aurora kinase can lead to a disorder of genetic material distribution, which can affect the stability of the genome [3]. In recent years, relevant studies have confirmed that Aurora kinase is highly expressed in a variety of tumor cells and participates in the formation of tumors [4,5]. Aurora A is a subtype of Aurora kinase. The Aurora A gene is located in the chromosomal region with active amplification. At present, many Aurora A compounds with different inhibitory activities have been found and show effective anticancer activities [6,7]. They involves a wide range of cancers, such as colorectal cancer, prostate cancer, ovarian cancer, breast cancer and glioma [8,9,10,11]. Therefore, Aurora A is considered to be a good marker of tumor progression and prognosis, and the inhibition of its kinase activity helps to reduce tumor invasiveness. (Figure 1 shows several kinds of Aurora kinase inhibitors entering clinical trials, including the first Aurora kinase inhibitor ZM-447439 (Figure 1(**1**)), the first Aurora B inhibitor, hesperidin (Figure 1(**2**)), and a new type of Aurora inhibitor, MLN-8237 (Figure 1(**3**)).

Despite the great importance of Aurora kinases in antitumor research, the specific processes and mechanisms of the involvement of Aurora kinases in tumor formation are still unclear. Due to safety concerns, there is still no marketed class of Aurora kinase inhibitors. Therefore, research on the more potent and safer class of Aurora kinase A inhibitor anticancer drugs is still challenging. A series of imidazo[4,5-*b*]pyridine derivatives have been reported to possess excellent potency as orally bioavailable Aurora kinase A inhibitors [12,13].

Quantitative structure-activity relationship (QSAR) is a common method of drug design. It is usually used as a common research method to explore the relationship between molecular structure and biological activity. Since its inception, QSAR has been widely used in drug design. The mechanism is to develop the relationship between chemical structure, physicochemical properties and biological activity of compounds in order to obtain a reliable statistical model for predicting the activity of new chemical entities [14]. The quantitative relationship between the chemical molecular structure and molecular biological activity is analyzed using a mathematical model. The quantitative relationship expression between activity and structure is established with molecular structure parameters as independent variables and molecular biological activity as dependent variables [15,16]. Based on the established model, through the use of virtual screening, molecular docking, ADMET, molecular dynamic simulation and other means, reasonable drug design can be carried out.

## 2. Materials and Methods

### 2.1. Dataset

The 3D conformations of all compounds in molecular construction are constructed using the SkechTool module in SYBYL2.0. The standard Tripos molecular force field and Powell energy gradient algorithm are used for energy optimization. All molecules are loaded with Gasteger-Huckel charges, the maximum number of iterations is 1000, the energy convergence limit is set to 0.005 kcal mol^−1^, and the other parameters adopt the system default values [17,18].

**Figure 2:**
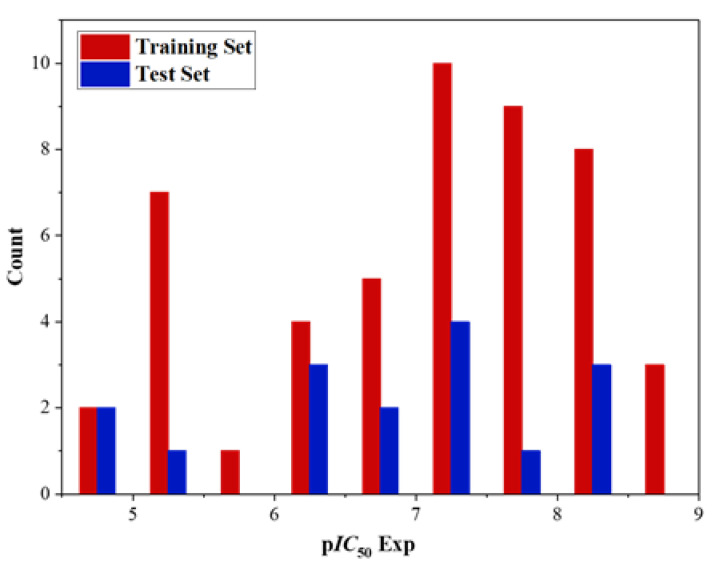
Distribution of experimental inhibitory activities (p*IC*_50_) for the training and test sets compounds in the QSAR models.

The dataset consisted of 65 imidazo[4,5-*b*]pyridine derivatives, all of which were synthesized and evaluated for biological activity by Vassilios Bavetsias and colleagues [19,20,21]. Compounds with similar structural framework and inhibitory ability were selected. The *IC*_50_ was the inhibitory activity of imidazo[4,5-*b*]pyridine derivatives against Aurora kinase A. For the convenience of calculation, these *IC*_50_ values were converted into corresponding p*IC*_50_ values to characterize their biological activities and used as the dependent variable of QSAR research. The conversion formula is: p*IC*_50_ = −lg *IC*_50_. In order to ensure the randomness of the data, we extracted 16 molecules from 65 molecules according to the ordinal number (one out of four) as the test set for external verification, and the other 49 molecules as the training set for model construction. The ratio of the test set to training set is 1:4. The main function of the training set is to build a QSAR model, and the test set is mainly used to externally verify the results of the QSAR model and evaluate the prediction ability of the model. In addition, when selecting the training set and test set, we should comprehensively consider the activity distribution of the whole molecular set, so as to ensure the effectiveness of the data and avoid its specificity (Figure 2). The structures and activities of all the compounds in the dataset are presented in Table 1.

### 2.2. HQSAR

Hologram Quantitative Structure-Activity Relationship (HQSAR) is a technique between 2D-QSAR and 3D-QSAR, which determines the relationship between biological activity and structural fragments and does not require molecular superimposition, 3D structure or choice of active conformation. It relies on 2D chemical database storage and the linear notation that defines the chemical structure, a process which involves the generation of fragments hash into an array called molecular holograms [22,23]. The ability of molecular alignment and conformational specification is achieved by converting the representation of the chemical structure of a molecule into the corresponding molecular hologram [24,25]. According to the principle of the holograms phase diagram, the effect of any group or atom in the drug molecule on the drug activity can be precisely determined, and the operation process is relatively fast [26].

The HQSAR method usually consists of several main steps. First, the molecule under study is cut into fragments of the appropriate size. The fragment structure is mainly determined by two parameters, Fragment Size and Fragment Distinction. Then, the encoded molecular fragment is converted into molecular holograms. Finally, after the molecular holograms are obtained, a quantitative model of the relationship between molecular holograms and compound properties is established by partial least squares regression.

In our study, the atoms of these fragments are encoded in holograms in different colors to reflect their contribution to biological activity, with negative, intermediate, and positive contributions represented in red, white and green, respectively. Each fragment is defined by its unique characteristic parameters, which are: atom (A), chemical bond type (B), atom connection (C), hydrogen atom (H), chirality (CH), and as donor or acceptor (DA). All the feature fragments of each molecule are mapped into a certain length integer string, the process is called molecular holography, and the length of integer string is called the molecular holographic length parameter. In HQSAR, the module provides 12 prime numbers (53, 59, 61, 71, 83, 97, 151, 199, 257, 307, 353 and 401) as a holographic length. Multiple HQSAR models with different predictive abilities can be obtained by combining different parameters. Therefore, we try to adopt as many combinations as possible to seek the optimal HQSAR model.

The method translates the molecular structure into characteristic molecular fingerprints, which consist of different types of molecular fragments and are labelled with 12 prime numbers (53, 59, 61, 71, 83, 97, 151, 199, 257, 307, 353 and 401). These labelled numbers were used as QSAR descriptors for modelling the biological activity prediction of the compounds. Meanwhile the HQSAR method combines different fragment descriptors such as atom (A), bond (B), linkage (C), chirality (Ch), hydrogen atom (H), donor and acceptor (DA), etc., and by combining different parameters, multiple HQSAR models with different predictive capabilities can be obtained. Therefore, in our study we consider both dimensionality and holographic length adjustments and try to use as many combinations as possible in order to build HQSAR models with good predictive ability.

### 2.3. CoMFA, CoMSIA and TopomerCoMFA

Comparative Molecular Field Analysis (CoMFA) is one of the most used methods to study the 3D-QSAR between drug and receptor. CoMFA can fully consider the 3D structural information of molecules, characterize the 3D field and static electric field through the molecular structure, conduct regression analysis of drug activity with these parameters as variables, and obtain the relationship between these 3D characteristic information and compound activities by partial least-squares method [27]. By comparing with the series of molecular space near the point of spatiality, by selecting a public skeleton to artificial molecule composite, electrostatic potential, and the physical and chemical parameters of molecules with biological activities can be calculated according to the molecular force field around the probe atoms. In this way, structure-activity relationship analysis of drugs can be achieved that can guide the design of the new compounds [28].

The Comparative Molecular Similarity Index Analysis (CoMSIA) method is better than the CoMFA method. In CoMSIA, the molecular similarity index calculated from the modified SEAL similarity field is used as a descriptor to consider simultaneously the spatial, electrostatic, hydrophobic, and hydrogen bond donor and hydrogen bond acceptor fields [29]. These indices are estimated indirectly by comparing the similarity of each molecule in the dataset to the common probe atoms located at the surrounding grid/lattice intersections [14]. The Gaussian-type function is used to describe the distance between probe atoms and atoms aligned with molecules in the dataset to calculate the similarity of all grid points [15].

Comparative molecular field analysis (CoMFA) and comparative molecular similarity index analysis (CoMSIA) are commonly used 3D-QSAR methods. Firstly, the compounds are compared using the distillation module implemented in SYBYL 2.0 to identify a common core scaffold in all molecules, and then based on the SYBYL molecular comparison, comparative molecular field analysis (CoMFA) and comparative molecular similarity index analysis (CoMSIA) studies are performed to explore the contribution of different interactions. The CoMFA and CoMSIA fields of each molecule are calculated for compounds in each network point computational dataset using a carbon probe hybridized with *sp*^3^ of charge +1.0, and then the relationship between the information of these three-dimensional features and the activity of the compounds is obtained by the partial least squares method [27]. In this way, the conformational relationship analysis of drugs can be achieved to guide the design of new compounds [28]. Specifically, the CoMFA method calculates spatial (S) and electrostatic (E) fields. In contrast to CoMFA, CoMSIA uses Gaussian functions and Gaussian-distributed similarity indices to avoid mutations in lattice-based probe-atom interactions [14,29], taking into account spatial, electrostatic, hydrophobic, and hydrogen-bond donor and hydrogen-bond acceptor fields [15].

Topomer Comparative Molecular Field Analysis (Topomer CoMFA) was proposed by Cramer et al. [16]. It is a 3D-QSAR tool which combines CoMFA and Topomer technology and can quickly predict the biological activities or properties of compounds. Topomer CoMFA method does not require manual superposition of molecules, can quickly and accurately predict the biological activity of compounds, and can build a relatively reliable model in a relatively short time [30]. Topomer CoMFA uses topological molecular technology to cut ligand molecules into two or more small fragments, while preserving the common skeleton as much as possible. It automatically builds a standard 3D model of topologies for each fragment and generates a set of spatial and static electric fields for each set of topologies. Finally, the parameters obtained are taken as independent variables and the biological activity value as dependent variables. The relationship between molecular structure and biological activity is described. Finally, the partial least square regression analysis method is used to establish the QSAR model [16,31,32].

In this study, in the CoMFA method, the aligned molecules with the best orientation are in the 3D cube lattice, with the grid spacing of 2.0 Å in the x, y and z directions, and an extension radius of 4.0 Å around the aligned molecules in the Cartesian direction. The Van der Waals radius and charge of *sp*^3^ hybrid carbon probe atom are 1.52 Å and +1.0, which are used to calculate the CoMFA space field and static electric field of each molecule. The Coulomb and Lennard-Jones potential functions are used to estimate the electrostatic and spatial interactions, respectively. The energy cut-off values for both the spatial field and the static field were set at 30 kcal mol^−1^. The CoMSIA method calculates the similarity index descriptor using the same lattice used in CoMFA. Five physical and chemical properties of spatial field, electrostatic field, hydrophobic field, hydrogen bond donor field and acceptor field were evaluated by Å probe atom with charge of +1.0, radius of 1 Å, hydrophobicity of +1.0, hydrogen bond donor field and hydrogen bond acceptor field, attenuation coefficient of 0.3 and grid spacing of 2.0 Å.

### 2.4. External Validation of the QSAR Model

The QSAR model usually adopts the Left-One-Out (LOO) method as the internal validation. The cross-validation coefficient *q*^2^ and non-cross-validation coefficient *r*^2^ are calculated through the formula as the basis for judging the quality of QSAR model [33]; see Formulas (1) and (2).

Among the parameters of the model, the larger the cross-validation coefficient *q*^2^ and non-cross-validation coefficient *r*^2^ mean, the better the correlation of the model and the stronger the prediction ability. Generally, the cross-validation coefficient *q*^2^ greater than 0.5, the non-cross-validation coefficient *r*^2^ greater than 0.6, and the difference between *r*^2^ and *q*^2^ less than 0.3 can prove that the model has high prediction ability [34,35]. Furthermore, $\hat{y_{i\;}}$ and $y_{i}\;$ is the predicted value and the experimental value of the test set, respectively, and $\bar{y}$ and $\hat{y\;}$ are the average activity values of the experimental value and the predicted value of the training set, respectively. The “*n*” is the number of molecules in the test set, and the “*i*” is the ordinal number of molecules in the test set.

(1) $$q^{2}=1-\frac{\sum_{i=1}^{n}{\left(y_{i}-{\hat{y}}_{i}\right)}^{2}}{\sum_{i=1}^{n}{\left(y_{i}-\bar{y}\right)}^{2}}$$

(2) $$r^{2}=\frac{{\left[\sum \left(y_{i}-\bar{y_{i}}\right)\left({\hat{y}}_{i}-\hat{y}\right)\right]}^{2}}{\sum {\left(y_{i}-{\bar{y}}_{i}\right)}^{2}\times \sum {\left({\hat{y}}_{i}-\hat{y}\right)}^{2}}$$

However, only the interior of the QSAR model validation parameters obtained through the LOO method does not directly determine the stand or fall of model. Good internal validation can only show that the values of cross-validation coefficient *q*^2^ and non-cross-validation coefficient *r*^2^ of compound training set are high, but it does not necessarily indicate that the prediction ability of the established model is good. Therefore, it is necessary to adopt external validation based on the test set [36].

External test set validation is an effective method to evaluate correction model prediction ability. Usually, the dataset is randomly divided into a training set and test set. The training set establishes a correction model, and the test set is used as an independent subset to test the prediction ability of the model. The external test set compares the predicted value with the experimental value to make an accurate evaluation of the prediction ability of the model.

In this study, the external validation of QSAR model can be verified and evaluated by the following parameters, and the prediction ability of QSAR model can be further verified by calculating the biological activity of compounds in the test set [37,38].

Prediction correlation coefficient $q_{pred}^{2}$ based on test set. $\sum_{i=1}^{n}{\left(y_{i}-\hat{y_{i}}\right)}^{2}$ is the sum of squares of the actual molecular activity of the test set and the mean molecular activity of the training set (SD). $\sum_{i=1}^{n}{\left(\hat{y_{i}}-y_{i}\right)}^{2}$ is the sum of squares of the deviation between the predicted value of the test set and the actual activity value (PRESS). Formula (3).

(3) $$r_{pred}^{2}=\frac{SD-PRESS}{SD}=1-\frac{\sum_{i=1}^{n}{\left(y_{i}-{\hat{y}}_{i}\right)}^{2}}{\sum_{i=1}^{n}{\left(y_{i}-\bar{y}\right)}^{2}}$$

Then, root mean square error (RMSE), mean determination error (MAE) and consistency correlation coefficient (CCC) were used to evaluate the performance of the regression model [39]. Formulas (4)–(6).

(4) $$RMSE=\sqrt{\frac{\sum_{i=1}^{n}{\left(y_{i}-{\hat{y}}_{i}\right)}^{2}}{n}}$$

(5) $$MAE=\frac{\sum_{i=1}^{n}\left|y_{i}-{\hat{y}}_{i}\right|}{n}$$

(6) $$CCC=\frac{2\sum_{i=1}^{n}\left(y_{i}-\bar{y}\right)\left({\hat{y}}_{i}-\hat{y}\right)}{\sum_{i=1}^{n}{\left(y_{i}-\bar{y}\right)}^{2}+\sum_{i=1}^{n}{\left({\hat{y}}_{i}-\hat{y}\right)}^{2}+n{\left(\bar{y}-\hat{y}\right)}^{2}}$$

In order to obtain the best prediction model for the test set, additional validation is performed on the model, where r and $r_{0}$ are the regression correlation coefficients between the actual activity value and the predicted activity value, respectively, and k and $k^{\prime }$ represent the slopes of the model. Other validation statistic parameters $r_{m}^{2}$ and $∆r_{m}^{2}$ are used to further evaluate the model. The parameters are as follows in Formulas (7)–(13) [40].

(7) $$r_{0}^{2}=1-\frac{\sum {\left(y_{i}-k\times {\hat{y}}_{i}\right)}^{2}}{\sum {\left(y_{i}-{\bar{y}}_{i}\right)}^{2}}$$

(8) $$r_{0}^{2^{\prime }}=1-\frac{\sum {\left({\hat{y}}_{i}-k^{\prime }\times y_{i}\right)}^{2}}{\sum {\left({\hat{y}}_{i}-\hat{y}\right)}^{2}}$$

(9) $$k=\frac{\sum \left(y_{i}\times {\hat{y}}_{i}\right)}{\sum {\left({\hat{y}}_{i}\right)}^{2}}$$

(10) $$k^{\prime }=\frac{\sum \left(y_{i}\times {\hat{y}}_{i}\right)}{\sum {\left(y_{i}\right)}^{2}}$$

(11) $$r_{m}^{2}=r^{2}\left(1-\sqrt{\left|r^{2}-r_{0}^{2}\right|}\right)$$

(12) $$r_{m}^{2^{\prime }}=r^{2}\left(1-\sqrt{\left|r^{2}-r_{0}^{2^{\prime }}\right|}\right)$$

(13) $$∆r_{m}^{2}=\left|r_{m}^{2}-r_{m}^{2^{\prime }}\right|$$

### 2.5. Virtual Screen

Virtual screening is a better method for the discovery of lead compounds in drug research and development. It plays a great role in the identification and optimization of lead compounds in the early stage of drug research and development. It can effectively reduce the cost of drug research and development and improve the speed of drug research and development [41]. In this study, we mainly used Topomer search technology in SYBYL2.0 for molecular virtual screening. Topomer search is a virtual screening method based on ligand drug design that can be used for structural optimization and skeleton transition of lead compounds [42]. Moreover, Topomer search technology can be used in combination with receptor-based molecular docking, and can also be used as a preliminary screening tool when there is no receptor structure [43].

Usually, the Topomer CoMFA model will give the contribution value of each R group fragment in each molecule. The contribution value of the R group fully combines the 3D field and electrostatic field of the Topomer CoMFA model to intuitively show the good or bad degree of each fragment in the form of numerical size. In most cases, the higher the contribution value of the fragment, the higher the activity of the whole molecule. The basic principle of Topomer search is based on the contribution value of small fragments given by the Topomer CoMFA model. Using the R group in the model as the question formula, through the comparison of Topomer similarity, search the molecular fragments with high similarity in the compound database [44], and then select the fragments with high activity contribution value. The reasonable combination of the searched fragments and the basic skeleton is used to optimize and transform the structure of the original compounds, so as to obtain compounds with high biological activity [45], so as to achieve the purpose of rational drug design.

### 2.6. Molecular Docking

Molecular docking studies the interactions between the design drugs based on the characteristics and their receptors. This is a theoretical simulation method to predict the binding pattern and affinity through the geometric, energy matching and recognition between ligand and proteins [46], which is an important technology in the field of computer-aided drug research. In this study, the Surflex-Dock module in the SYBYL2.0-X software package is selected to perform the simulated docking [47]. Surflex-Dock is a semi-flexible docking with rigid protein structure, which uses a Protomol-based method to dock the ligand into the binding site of the receptor. It can avoid the tedious process of searching for the active site, and can compile specific docking methods, extract the ligands bound at the original site to generate a docking protocol, and dock the target ligand into the previous specific site. In addition, the conformations of the generated ligand molecules are compared with the original ligands, and are used as a reference to determine whether the docking method is applicable [48]. The docking results of original ligands can also be compared with molecules of potential ligands to further screen drugs that are more suitable as inhibitors.

The protease used for this docking was derived from the PDB database (Protein Data Bank) [49], with the ID 1MQ4. The 1MQ4 macromolecular protein was pretreated before performing molecular docking to remove the required small molecule ligand from the macromolecular complex, the unwanted small molecule ligand and all water molecules, to hydrogenate the protein, and to add Gasteiger-Huckel charges [50,51]. The docking regions were determined by analyzing the interactions of ligands and active residues, and the active sites for docking were determined according to the small molecule ligands.

The docking results were judged by the values of the compounds’ scoring functions total score, crash and polar. The score function value of total score indicates the affinity between small molecule ligands extracted from macromolecular proteins and receptors, and the higher value means higher affinity. The crash represents the incompatibility between ligands extracted from macromolecular proteins and receptors, and the smaller crash value indicates the more compatible of molecular docking. Polar is the score of polarity function. A higher value is expected to be obtained when the binding site is on the protein surface, and a smaller value is expected when the binding site is inside the protein [52,53].

### 2.7. Molecular Dynamics Simulation

To explore the structural and energetic status of the newly designed compound, molecular dynamics (MD) simulation of 50 ns was performed using GROMACS 2020.4 software. The protein topology generation was used CHARMM36-February 2021 force-field, the ligand topology and the force field parameter files were generated from the CHARMM General Force Field server (CGenFF). These topology files were merged in GROMACS. Subsequently, the protein-ligand system was placed in a 12-sided box composed with a Tip3p water model. To neutralize the system charge, six Cl^−^ were added to the system. Then the entire system was minimized by the steepest drop minimization algorithm. Following this, a 50 ns simulation was run under NPT (or NVT) ensemble at constant temperature (300 K) and pressure (1 atm) with the integration of trajectories at every two femtoseconds (fs), the atomic coordinates of simulated structures were recorded at every 10 ps. Root mean square deviation (RMSD), root mean square fluctuation (RMSF), radius of gyration (Rg) and number of hydrogen bonds were analyzed over the full trajectory. Furthermore, the free energy landscapes (FEL) were described to verify the stability of newly designed compound in complex with 1MQ4 protein.

The protein-ligand binding free energies were calculated by Molecular Mechanics-Poisson Boltzmann Surface Area (MM/PBSA). The last 10 ns of all MD simulations trajectories were subjected to calculate the binding free energy through MM/PBSA analysis. For calculations, 100 snapshots were extracted at every 100 ps from the last 10 ns of MD trajectories. Herein, the calculation of binding free energy ΔG_bind_ was performed using the following equation:

(14) $$∆G_{bind}={E_{vdw}+E}_{elec}+E_{polar}+E_{SASA}-TS$$

E_vdw_ means the Van der Waals energy, E_elec_ means electrostatic energy, E_polar_ means electrostatic of salvation, and E_SASA_ means the non-electrostatic energy of salvation. In addition, T is the temperature, S is the entropy, and TS is the entropic contribution in a vacuum. Because the variation in TS terms does not improve the anticipated results, the TS term is ignored.

### 2.8. ADMET Prediction

Currently, the research and development of drugs is a high-risk investment, which often faces unexpected and even catastrophic failures at different stages of drug discovery [54]. One of the main reasons for the failures is the lack of efficacy and safety, which is related to the absorption, distribution, metabolism and excretion (ADME) properties and various toxicities (T) of the human body. Rapid ADMET evaluation to reduce failures in drug discovery is essential [55]. ADMET (Absorption, Distribution, Metabolism, Excretion, and Toxicity) are prerequisites, and the nature of the molecule plays a key role in the preclinical phase. It is therefore necessary to pretest the ADMET properties of the designed compounds to ensure drug suitability for the human body [56]. The ADMET properties acquired in this study are derived from the web flat ADMET-lab [57].

## 3. Results and Discussion

### 3.1. The Data Analysis

#### 3.1.1. Result Analysis of HQSAR Model

The HQSAR model can be optimized by changing the parameters, including holographic length, Fragment Size and fragment characteristic parameters. In this article, the hologram length was the default (97, 151, 199, 257, 307, 353, and 401) and the Fragment Size set as the default (4–7). Different HQSAR models were established for 49 compounds in the training set using different fragment types. According to the combination of different fragmentary features, the best 32 combination methods selected are presented in Table 2.

As shown in Table 2, the best model could be obtained when the Fragment Distinction set to “A/CH” with the condition of all Fragment Size of “4–7” (Model 1-07), whose main parameters *q*^2^ = 0.872, *r*^2^ = 0.961, N = 6, HL = 151. Based on the results of Table 2, the Fragment Dispersion is set as “A/CH” again, with different Fragment Size chosen, 10 HQSAR models are built and shown in Table 3. The results show that the best model can be obtained when the Fragment Size is set as “2–4”. The main parameters are shown as *q*^2^ = 0.892, *r*^2^ = 0.948, N = 5, HL = 257. Therefore, the best HQSAR model (Model 2-03) is built when the Fragment Dispersion is “A/CH” and the Fragment Size is “2–4”.

The HQSAR results could be used to discover the potential effects of fragments and atoms on the activity of compounds, which is helpful to reveal the molecular mechanism affecting the activity of compounds. From the color code diagram, fragments and atoms that might be the key contributors for the activity of Aurora kinase inhibitors can be identified. Among them, the color code from green to red represents the activity contribution from high to low.

**Figure 3:**
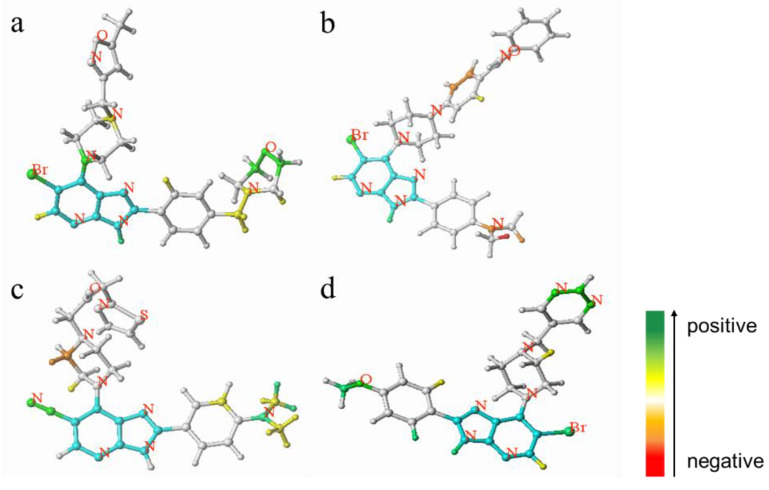
Atomic contribution maps of compound **52** (**a**), compound **32** (**b**), compound **21** (**c**) and compound **40** (**d**). (Cyan indicates common backbone, green or yellow indicates positive contribution, orange and red indicate negative contribution, and white indicates intermediate contribution to biological activity).

The color code diagram of compound **52** (p*IC*_50_ = 8.70) and compound **40** (p*IC*_50_ = 8.10) in the HQSAR model are shown in Figure 3a,d. The multiple green and yellow fragments indicate that these fragments and atoms contribute positively to biological activity and should be retained when synthesizing compounds with greater biological activity. The HQSAR contribution diagrams of compound **32** (p*IC*_50_ = 6.56) and compound **21** (p*IC*_50_ = 7.30) are shown in Figure 3b,c. There are many red fragments in the figure, which indicated negative contributions to biological activity of these fragments and atoms. The atoms of the other substituents in the compounds are white, which indicates that they have neutral contributions to biological activity and can replaced by substituents with stronger inhibitory activities.

#### 3.1.2. Result Analysis of CoMFA and CoMSIA Models

Generally, in the process of research, the molecule with the highest p*IC*_50_ value in the whole molecule set has the best molecular structure and is suitable to be used as the template molecule. Through the analysis of template molecules, the relationship between molecular structure and biological activity can be found quickly and accurately. Structure alignment is one of the most important input variables in 3D-QSAR analysis, and the reliability of the contour map based on molecular structure alignment greatly influences the predictive ability of the model. In this study, compound **52** with the highest activity is suitable for analysis as a template, and the common skeleton is selected as the overlap site.

As shown in Table 4, for the CoMFA model, the optimal composition number was 6, *q*^2^ was 0.866, *r*^2^ was 0.983, SEE was 0.156, and F was 403.587. This proves that this established model could strongly and stably predict the activity value of test set compounds. The contribution of spatial field and static electric field were 48.6% and 51.4%, respectively, which indicates the contribution of electric field to the CoMFA model is relatively large.

For theCoMSIA model, the results of the CoMSIA model are listed in Table 4 from 3-01 to 3-30. Model 3-30 is chosen as the research object for further discussion and analysis. The optimum composition was 9, *q*^2^ was 0.877, *r*^2^ was 0.995, SEE was 0.091, and F was 802.161. The contribution values of space field, electrostatic field, hydrophobic field, hydrogen bond donor field and hydrogen bond acceptor field were 18.3%, 31.6%, 22.1%, 8.9% and 19.2%, respectively. The results show that the contribution value of electrostatic field to CoMSIA model is larger.

**Figure 4:**
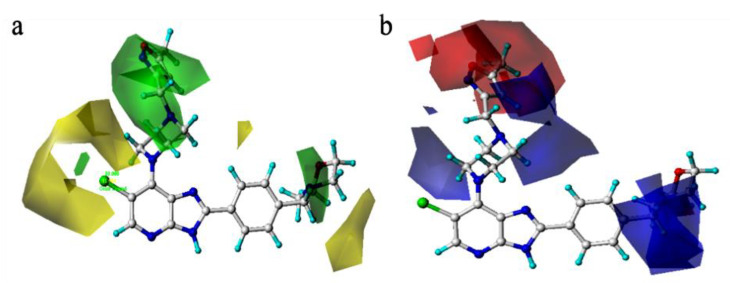
Three-dimensional (3D) (**a**)spatial field and (**b**)static electric field action diagrams for the CoMFA model with compound **52** as the template molecule.

Figure 4 shows the 3D contour map of the spatial field and static electric field of the CoMFA model of template molecule compound **52**. The green part indicates that increasing the volume of substituents is beneficial to the improvement the compound activity, while the yellow part indicates that reducing the volume of substituents is beneficial to the improvement of compound activity. The red area indicates that increasing the electronegativity of the group would increase the activity of the compound, and the blue area indicates that decreasing the electronegativity of the group would increase the activity of the compound. In Figure 4, the proportion of the green group and blue group are obviously larger. In order to improve the molecular activity, it is necessary to increase the volume of substituents in the corresponding parts or reduce the electronegativity of substituents in the blue region.

**Figure 5:**
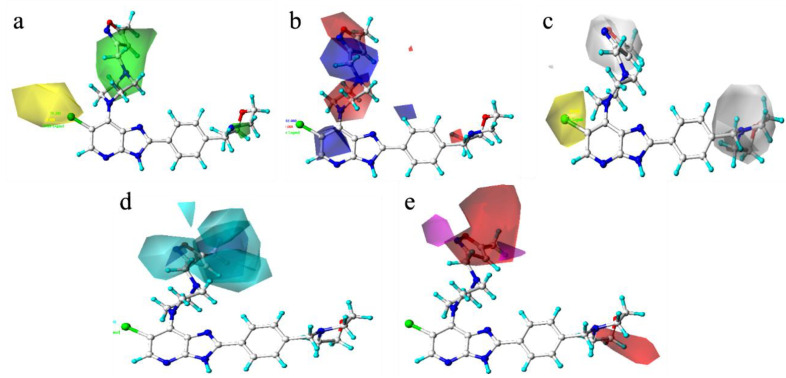
Three-dimensional (3D) contours of the CoMSIA model with compound **52** as template ((**a**): spatial force, (**b**): electrostatic force, (**c**): hydrophobic force, (**d**): hydrogen bond donor force, (**e**): hydrogen bond acceptor force).

Figure 5 shows the 3D contour map of CoMSIA model of compound **52**. In Figure 5a, the green part indicates that increasing the volume of substituents is beneficial to improve the activity of the compound, and the yellow part indicates that reducing the volume of substituents is beneficial to improve the activity of the compound. In Figure 5b, the red region indicates that increasing the electronegativity of the group is beneficial to improve the activity of the compound, and the blue region indicates that reducing the electronegativity of the group is beneficial to improve the activity. In Figure 5c, the white part indicates that the increase of hydrophilic group is beneficial, and the yellow part indicates that the increase of hydrophobic group is beneficial. In Figure 5d, the cyan part indicates that increasing hydrogen bond donor is beneficial, and the purple part represents increasing hydrogen bond donor is not favorable. In Figure 5e, the magenta part indicates that increasing hydrogen bond receptor is beneficial, while the red part indicates that increasing hydrogen bond receptor is not conducive to activity. According to the 3D contour map of CoMSIA model, we can increase or decrease specific groups in corresponding parts to improve the activity of compounds.

#### 3.1.3. The Result and Analysis of the Topomer CoMFA Model

**Figure 6:**
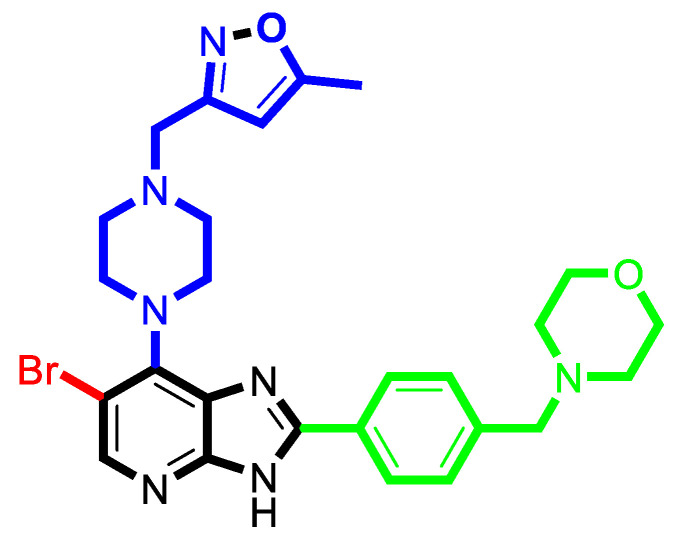
Schematic representation of segmentation methods and fragments using compound **52** as a template molecule.

In the Topomer CoMFA study, the most active compound (**52**) was analyzed as a template, and all the compounds were divided into four fragments according to the segmentation method shown in Figure 6. After cutting, the compounds were automatically divided into R_a_ group (red), R_b_ group (blue), R_c_ group (green) and common skeleton (black).

Taking compound **52** as the template molecule, the relationship between the structure and activity of the compounds in training set was studied using the Topomer CoMFA method. The results are shown as follows:

In Table 5, *q*^2^ of the best model obtained is greater than 0.5, *r*^2^ greater than 0.6, and residual between *r*^2^ and *q*^2^ less than 0.3. This indicates that the model established by this method is an ideal Topomer CoMFA model, and its statistical results have high predictive ability. At the same time, the predicted values of SEE, *q*^2^ stderr, *r*^2^ stderr and F are 0.199, 0.36, 0.20 and 369.402, respectively. These results indicate that the error of the model is quite small, which further demonstrates that the established model is reliable.

**Figure 7:**
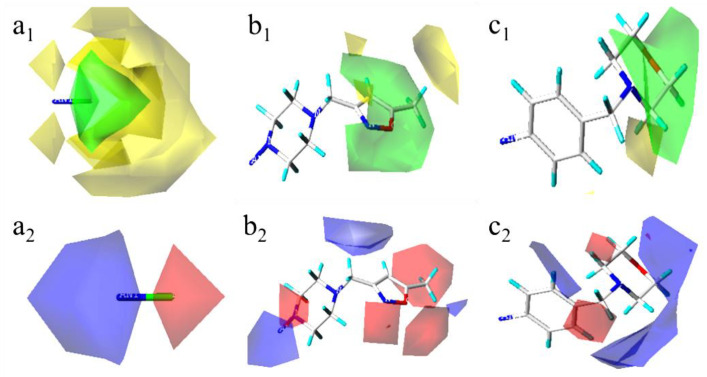
Three-dimensional (3D) contour map of the Topomer CoMFA model based on template **52**. (**a_1_**) the spatial contour map of R_a_ group; (**a_2_**) the electrostatic contour map of R_a_ group; (**b_1_**) the spatial contour map of R_b_ group; (**b_2_**) the electrostatic contour map of R_b_ group; (**c_1_**) the spatial contour map of R_c_ group; (**c_2_**) the electrostatic contour map of R_c_ group.

Figure 7 shows the 3D contour map of the Topomer CoMFA model of the template molecule compound **52**. Figure 7a_1_,a_2_ are the spatial contour map and electrostatic contour map of R_a_ group, respectively. Figure 7b_1_,b_2_ are the spatial contour map and electrostatic contour map of R_b_ group, respectively. Figure 7c_1_,c_2_ are the spatial contour map and electrostatic contour map of R_c_ group, respectively. In Figure 7, the green part indicates that increasing the volume of substituent is beneficial to improve the activity of the compound, and the yellow part indicates that reducing the volume of substituent is beneficial to improve the activity of the compound. The red region indicates that increasing the electronegativity of the group is beneficial, and the blue region indicates that reducing the electronegativity of the group is beneficial.

### 3.2. Comparison of HQSAR, CoMFA, CoMSIA and Topomer CoMFA Models

The comparisons of the predicted activity values obtained by HQSAR, CoMFA, CoMSIA and Topomer CoMFA models and the residual values obtained by subtracting the experimental values from the predicted values are listed in Table 6. The activities of these compounds predicted by the four methods are basically the same as the experimental values, which indicates that the results of the model established by the four methods are accurate and have a high predictive ability.

Based on the results of training set, the p*IC*_50_ values of the test set are predicted by correspond models and the residual values are also calculated (Table 6). According to the contents of Table 6, the linear regression analysis diagram (a_1_) and residual analysis diagram (a_2_) of the HQSAR model, the linear regression analysis diagram (b_1_) and residual analysis diagram (b_2_) of the CoMFA model, the linear regression analysis diagram (c_1_) and residual analysis diagram (c_2_) of the CoMSIA model and the linear regression analysis diagram (d_1_) and residual analysis diagram (d_2_) of the Topomer CoMFA model are drawn using Origin Pro (edu) software. In these figures, all samples in the linear regression diagram are evenly distributed around the 45° line, and the data in the residual analysis diagram are mainly concentrated around the 0-tick line. This demonstrates that all four models built in this study have favorable predictive ability.

**Figure 8:**
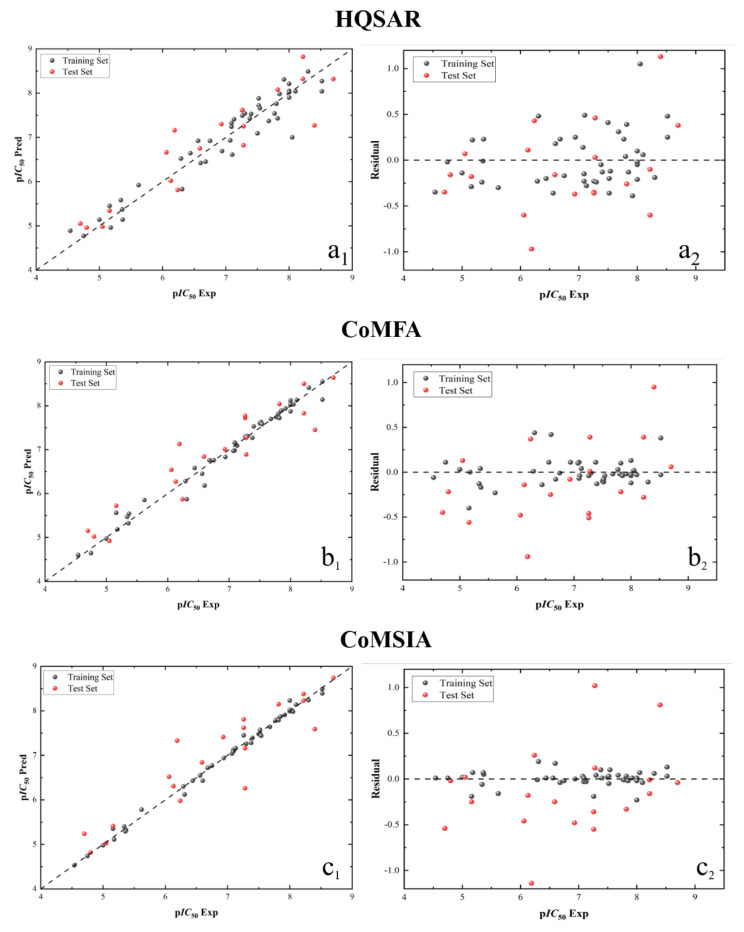

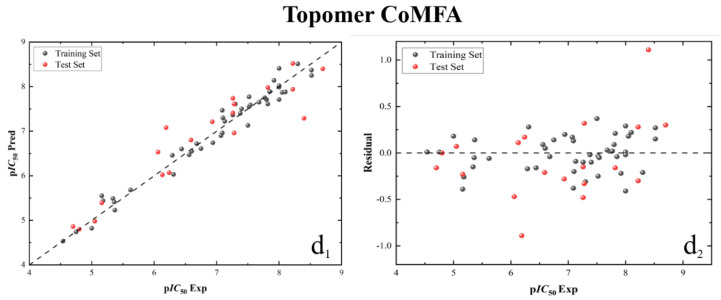
Linear regression analysis diagram and residual analysis diagram of HQSAR (**a_1_**,**a_2_**), CoMFA (**b_1_**,**b_2_**), CoMSIA (**c_1_**,**c_2_**) and Topomer CoMFA (**d_1_**,**d_2_**) model results.

By comparing and analyzing the prediction results of HQSAR, CoMFA, CoMSIA and Topomer CoMFA models (Table 6) with the linear regression and residual analysis diagram (Figure 8), the predicted values obtained by the four established models are quite similar to the experimental values. This indicates that the Fragmentation Interval and Size in the HQSAR model, the overlapping position of CoMFA and CoMSIA model, and the choice of cutting method in the Topomer CoMFA model are all the optimum. Therefore, the model established in this study could be used to predict the biological activities of these types of new compounds and their derivatives more accurately.

### 3.3. External Validation Results of the QSAR Model

Through the analysis and comparison of the models built by QSAR, good results are achieved. However, these results were theoretically tested based on the training set, which might be accidental. In order to further verify the predictive ability of QSAR model, independent test sets not used for model generation are used for further analysis (Table 7).

In Table 7, combined with the relevant parameters and corresponding standards introduced above, the results of external verification of the four models are basically within a reasonable range, which further shows that the model established in this study has high reliability.

### 3.4. The Result and Analysis of Molecular Design

Based on the analysis of the Topomer CoMFA model results, suitable R_a_, R_b_ and R_c_ groups are designed based on the template compound **52**. Taking the R_a_, R_b_ and R_c_ groups as the query structures, the Topomer search technology is used to search the conformation in the ZINC database. Among the structural fragments, the R_a_ group (contribution value is 0.80), R_b_ group (contribution value is 1.54) and R_c_ group (contribution value is 1.77) with topological distance close to 185 and activity contribution value higher than the template molecule are selected. Finally, 10 new imidazo[4,5-*b*]pyridine derivatives are designed and screened as Aurora kinase A inhibitors. According to the previous method, the structure of the new compound is constructed in SYBYL2.0-X, and the molecular optimization and naming are carried out by the same method. The activities of newly designed compounds are predicted by Topomer CoMFA model as shown in Table 8.

As shown in Table 8, the predicted activity values (p*IC*_50_) of all the newly designed compounds are higher than the template molecules (p*IC*_50_ = 8.70). The structural analysis of the newly designed compound molecules are carried out. For R_a_ group, when the -CN group is replaced by -Br group, the volume of the substituent group is increased and the electronegativity is enhanced. The corresponding criteria are that enlarging the volume around the green contour is favorable to the activity of inhibitors (Figure 8a_1_,a_2_). Increasing the electronegativity around the red group is conducive to the increase in activity (Figure 8b_1_,b_2_). For the R_b_ group, the newly designed compounds increased the volume near C-33 of the Rb group, and C-36 position is deleted, corresponding to the principle that increasing the volume around the green group and reducing the volume around the yellow group are beneficial to increase activity. These results are consistent with the analysis results of the QSAR models.

### 3.5. Analysis of Molecular Docking Results

**Figure 9:**
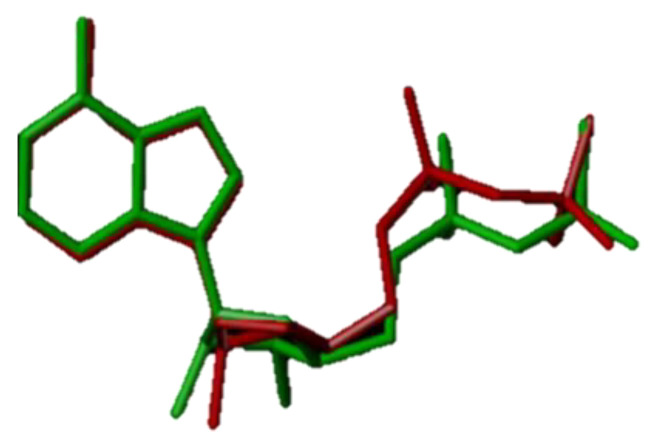
Conformational superposition of docked ligand and extracted primary ligand (ADP). (The red bars in the figure represent the docked ligand and the green bars are the original ligand).

In order to understand the inhibitory protein mechanism of the designed compound and the binding between the newly designed compound and the receptor protein, it was necessary to perform the molecular docking. According to the three probes NH, CH_4_, and CO, which form binding pockets with small molecule ligands, the three probes represent the hydrogen bond donor, hydrogen bond acceptor, and hydrophobic sites, respectively. The cocrystal ligand in protein macromolecules is then re-accessed into the crystal structure by docking technique to observe its docking in the interface pocket to verify the reliability. The ligand molecules before docking are contrasted with those after docking (Figure 9). The red sticks in the Figure represent the ligands after docking, and the green sticks are the original ligands. The conformations of crystal ligands almost completely overlap with those after ligand docking (similarity of 0.903), and their rotation tendency is basically similar. According to Figure 9, the docking conformation of ligand molecules basically matched the original conformation, and the docking method in this study is reasonable and reliable.

Molecular docking was necessary to understand the mechanism of protein inhibition by the designed compounds and the binding of the newly designed compounds to the receptor proteins. The eutectic ligand (ADP) in the protein macromolecule was rewired into the crystal structure by docking technique, and the ligand molecules before docking were compared with the ligand molecules after docking (Figure 9). The red bars in the figure represent the docked ligand and the green bars are the original ligand. The conformation of the crystalline ligand overlaps almost completely with that of the ligand after docking (similarity of 0.903), and their rotational trends are essentially similar. As can be seen from Figure 9, the docked conformations of the ligand molecules basically match the original conformations, and the docking method in this study is reasonable and reliable.

The template molecule and all the newly designed compounds are put into the docking pocket for molecular docking, and the template molecule is used as a control, with the value of the scoring functions, total score, crash and polar, and the number of hydrogen bonds formed as the criteria. A higher value of the total score indicates that the docking results meet the requirements of the analysis results (Table 9).

**Figure 10:**
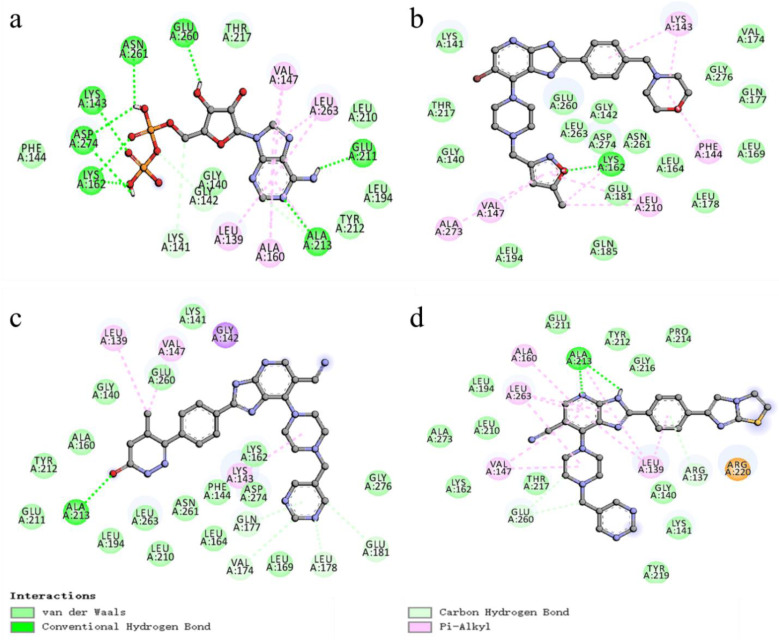
Two-dimensional (2D) view of the binding conformation and interactions within the protein active site (PDB: 1MQ4). (**a**) original ligand ADP (**b**) compound **52** (**c**) compound N1 (**d**) compound N2.

In the 2D analysis diagram of molecular docking (Figure 10), the ball-and-sticks are ligands, and the round spheres are the amino acid residues forming interactive forces. Hydrogen bonding is shown as the green dotted line, and hydrophobic interaction is shown as the pink dotted line. Among them, the hydrogen bond is the main force to maintain the ligand molecules of protein and compound, which made the binding between them more stable. The hydrophobic bonds are mainly used to enhance the affinity between ligand and amino acids around active site.

Figure 10a shows a docking analysis diagram of ligand extracted from 1MQ4 protein crystals. In this Figure, the ligand mainly forms four hydrogen bonds with A/ASN261, A/GLU260, A/ASP274, A/GLU211 and A/ALA213 residues in protein crystals, and hydrophobic interactions formed with amino acid residues such as A/VAL147, A/ALA160, A/LEU263 and A/LEU139. Total score, crash and polar are 9.3093, −0.5178 and 1.0743, respectively. The selection of ligands and protein crystals used in this docking is appropriate, and the docking method is reasonable and reliable.

Figure 10b shows the 2D analysis diagram of the docking 1MQ4 protein crystal with compound **52** in the training set as the template molecule. Compound **52** mainly forms a hydrogen bond with A/LYS162 residue in the crystal structure, and forms a hydrophobic interaction with amino acid residues such as A/LYS143, A/PHE144, A/LEU210 and A/VAL147. The total score, crash and polar are 6.3356, −0.5557, and 1.1914, respectively. Figure 10c shows that the newly designed compound N1 forms four hydrogen bonds with A/GLN177, A/ALA213, A/VAL174 and A/GLU181 residues, and forms a hydrophobic interaction with amino acid residues such as A/LEU139, A/VAL147 and A/LYS143, with total score, crash and polar as 6.3210, −1.7537 and 1.2747, respectively. Figure 10d shows that the newly designed compound N2 forms two hydrogen bonds with A/ALA213, A/ARG137 and A/THR260 residues, and forms a hydrophobic interaction with amino acid residues such as A/ALA160, A/LEU263, A/VAL147 and A/LEU139. The total score, crash and polar are 7.8842, −1.4843 and 2.2739, respectively.

The results show that the newly designed compounds formed strong hydrogen bonds with amino acid residues such as A/ALA213, A/VAL174 and A/LEU263 and forms hydrophobic interactions with amino acid residues such as A/VAL147, A/LYS143 and A/LEU263. These interactions enhance the binding strength of ligands and receptors, and their scoring functions are all ideal. Thus, the designed compound docking results are reliable.

### 3.6. The Analysis of Molecular Dynamics Simulation

MD simulation analysis was used to study the nature of newly designed compound in dynamic states and the stability of the protein-ligand complex. All molecular dynamics simulations were carried out for template **52**, N3, N4, N5 and N7 docked with 1MQ4 protease structure for 50 ns. Meanwhile, the interaction between compounds N3, N4, N5, and N7 with the receptor protein is depicted in Figure S1.

**Figure 11:**
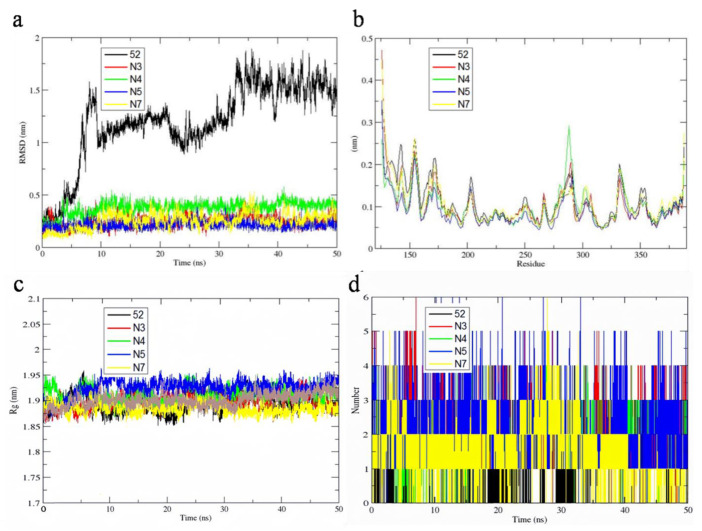
RMSD values of ligands at the active site of 1MQ4 during 50 ns MD simulation (**a**). The RMSF values of chain A in five protein-ligand complexes (**b**). Radius of gyration values in five protein-ligand complexes (**c**). The hydrogen-bond numbers between 1MQ4 and the ligands (**d**).

The RMSD is one of the pivotal parameters which can explain the overall stability and describe its structural conformation changes in the protein-ligand complex from MD simulation trajectories. The high stability of the complex can be obtained by the low and stable RMSD value. Figure 11a represents the RMSD plot for the original ligand and newly designed compounds (N3, N4, N5, N7) docked with 1MQ4 protein. Compared to the template ligand-protein complex, the RMSD fluctuation of new compounds system became progressively lower. After 40 ns, the RMSD fluctuation reaches the minimum and equilibration is expected to occur. This suggests the system of newly designed compound combined with 1MQ4 protein is more stable.

The mean and standard deviation values of RMSD and Rg of every complex are shown in Table S1.

The RMSF study confirms the flexible regions of the protein and determines the fluctuation of protein during the MD simulation. A higher RMSF value indicates that the atoms in the molecule have a greater change in position during the simulation, suggesting that these atoms are more flexible. On the contrary, a lower RMSF value indicates that the atoms have less positional changes, suggesting that these atoms are more stable during the simulation. Figure 11b shows the fluctuations of the residue of newly designed compounds (N3, N4, N5, N7) docked with 1MQ4 protein. In the region of 175–190 and 210–270, the fluctuations of RMSF values are smaller, which is because the key residues, such as A/GLU181, A/ALA213, A/LEU263 and A/THR260 at the active sites of protein 1MQ4 are more stable than other residues during the whole MD simulation process. These results are consistent with the molecular docking results. Furthermore, from the lower RMSF value, designed compounds N3, N5 and N7 showed better stability with protein 1MQ4.

The radius of gyration (Rg) is calculated to describe the compactness changes in the system and the stability of the system. It denotes the folding and unfolding of the proteins during MD simulations. The values of Rg for newly designed compounds are shown in Figure 11c. Five complexes show stable compactness and radius of gyration. These results indicate the compounds designed are superior in stability.

The hydrogen bonds between protein and ligand are important factors in maintaining the stability of complex conformations. Figure 11d shows the number of hydrogen bonds of five protein-ligand complexes during the 50 ns simulation. The number of hydrogen bonds varies for N3 (0–6), N4 (0–5), N5 (0–6) and N7 (0–6). The averages of the hydrogen bonds are calculated for 0.66 (template **52**), 2.21 (N3), 1.84 (N4), 2.21 (N5) and 1.51 (N7). These results show that these newly designed compounds are more stable than template compound **52** during the simulation process.

**Figure 12:**
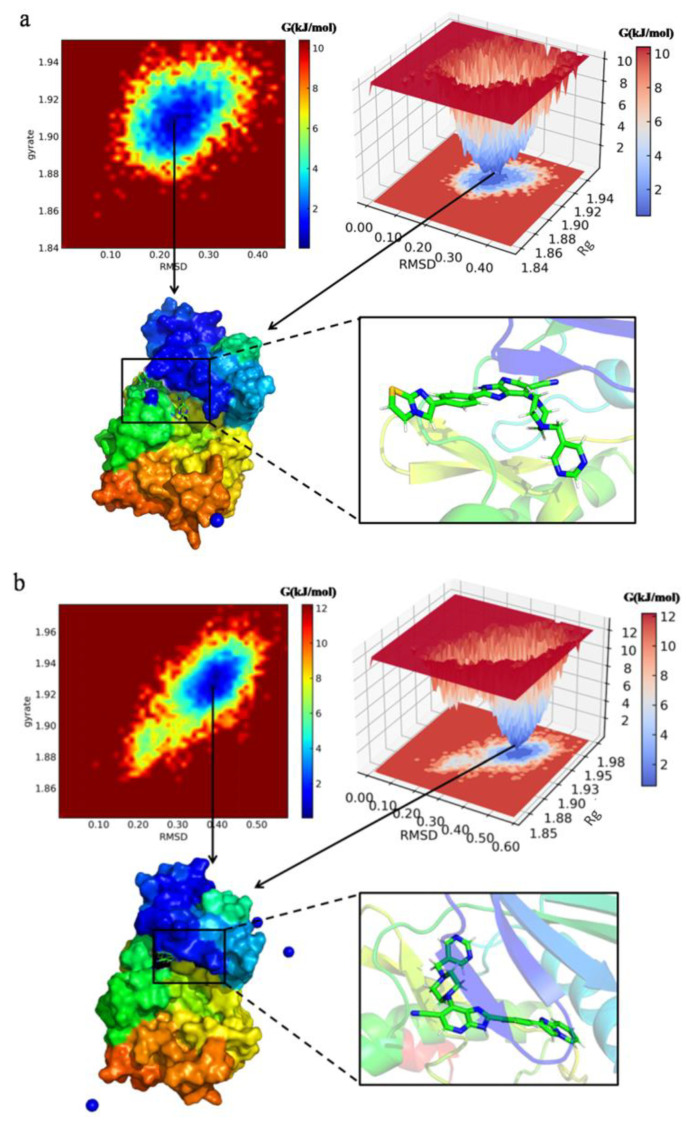

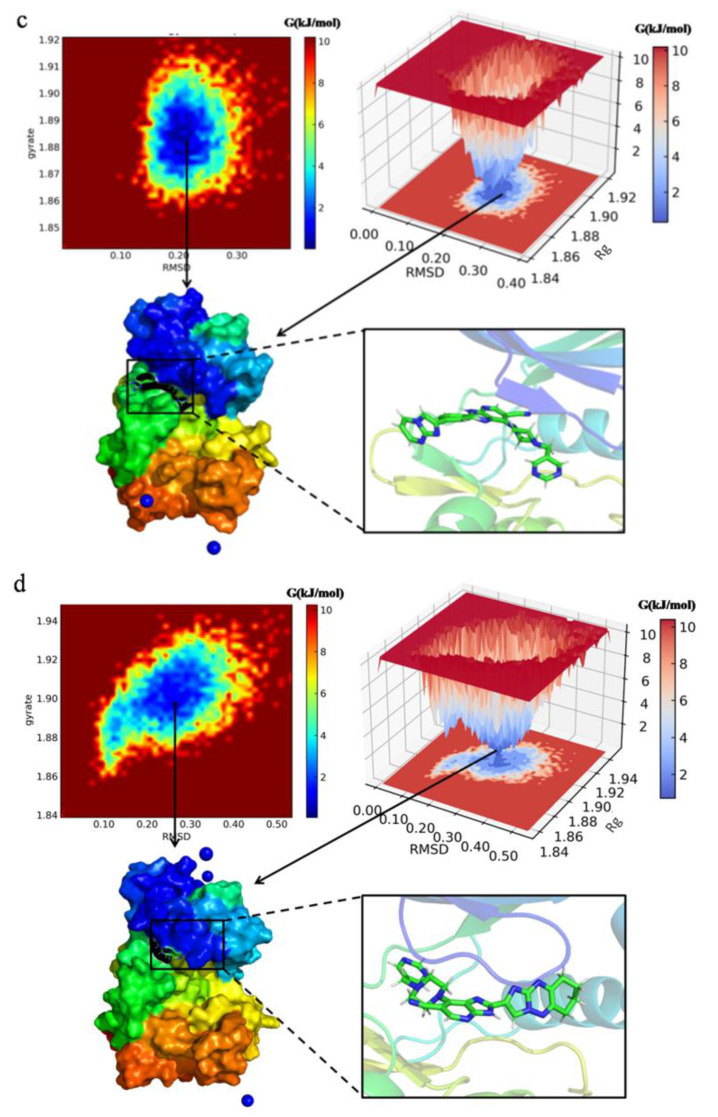
Free energy landscape (FEL) plotted between RMSD and Rg coordinates, snapshot from lowest energy conformational state of the newly designed compounds ((**a**): N3, (**b**): N4, (**c**): N5, (**d**): N7) in complex with protein 1MQ4. The colored scale plot shows the free energy profile (kJ/mol), the red color region in plot represents the metastable state, and the dark blue region represents the lowest energy state.

The free energy landscape is computed to characterize the conformational changes in the four protein-ligand complexes during the MD simulation. Stability is color-coded from dark blue to red. The free energy landscapes plotted between RMSD and Rg coordinates for N3, N4, N5 and N7 are displayed in Figure 12 with the Gibbs free energy varying from 0 to 9.4 kJ/mol, 11.2 kJ/mol, 9.2 kJ/mol and 9.4 kJ/mol, respectively.

As shown in the free energy landscape, the ensemble of conformations of the newly designed compounds (N3, N4, N5, N7) in complex with protein 1MQ4 are restricted into a single conformation cluster and present favorable basins. Multiple favorable free energy basins of N3 in complex with protein 1MQ4 are formed at RMSD values fluctuating between 0.18 and 0.31 nm, and Rg values varying between 1.89 and 1.92 nm. The free energy basins of N4 in complex with protein 1MQ4 are located at RMSD values between 0.32 and 0.45 nm, and Rg values varying between 1.91 and 1.94 nm. The free energy basins of N5 in complex with protein 1MQ4 are located at RMSD values between 0.18 and 0.25 nm, and Rg values varying between 1.87 and 1.90 nm. The free energy basins of N7 in complex with protein 1MQ4 are relatively scattered, and located at RMSD values fluctuating between 0.20 and 0.35 nm, and Rg values varying between 1.88 and 1.92 nm. These free energy profiles clearly correlate with the previous RMSD and Rg analysis. The surface formats and snapshots of lowest-energy conformation from free energy basins are extracted and represented in Figure 12, which depict the stable conformational states of the complexes.

The Van der Waals energy (*E*_vdw_), electrostatic energy (*E*_elec_), polar solvation energy (*E*_polar_), SASA energy (*E*_SASA_) and binding free energy (*G*_bind_) are calculated and listed in Table 10. The results show that the binding free energies for N3, N4, N5 and N7 complex structure are −68.515 ± 2.218, −54.869 ± 1.705, −62.317 ± 2.418 and −52.857 ± 4.201 kcal/mol whereas that of template compound **52** complex are −26.939 ± 5.302 kcal/mol. The negative values represent the binding interaction, while the positive values represent the opposite. The newly designed compounds show negative values which strongly suggest that these compounds bind with Aurora protein (1MQ4) efficiently and provide a direction for further research.

### 3.7. The Analysis of ADMET Prediction Results

From the results of ADMET property prediction in Table 11, it is clear that all the new compounds showed better human intestinal absorption (HIA), most of them had high oral bioavailability (F) and blood-brain barrier (BBB) distributions, but the plasma protein-binding (PPB) indices were comparatively low, so that the dose of the drug needs to be taken into account when designing the drug. Inhibitory capacity prediction showed that all compounds could act as substrates for CYP3A4 and CYP2C9 inhibitors. Drug clearance prediction showed that most of the compounds exhibited moderate clearance (5–15 mL/min/kg). On this occasion, compound toxicity prediction assessment revealed that all compounds passed Ames toxicity and were not carcinogenic in rats, which is a positive feature for drug development. In conclusion, the ADMET prediction results suggest that the new compounds have potential medicinal value for drug development. However, they still need to pass rigorous testing to ensure drug safety and efficacy that can be guaranteed in subsequent studies and clinical trials.

Based on the results of the model built by four methods, HQSAR, CoMFA, CoMSIA and Topomer CoMFA, virtual screening is performed using Topomer search technology, and 10 novel compounds belonging to the Aurora kinase A inhibitor class are designed based on the screening results. However, the pharmacology and safety of the designed compounds are uncertain. It is necessary to pretest the ADMET properties of the designed compounds to ensure drug suitability in humans.

The results are shown in Table 11. Each value of the designed drugs basically meets the requirement without rat carcinogenicity, which indicates that the designed drug molecules have high safety and obvious pharmacological effects.

## 4. Conclusions

Cancer is a major disease that seriously threatens human life and social development. Utilizing scientific methods for cancer prevention and control has become one of the most important global public health issues. This study established reliable 3D-QSAR models based on 65 imidazo[4,5-*b*]pyridine derivatives, analyzing the relationship between inhibitor bioactivity and structure. It designed novel imidazo[4,5-*b*]pyridine-based Aurora kinase A inhibitor anticancer drugs, ultimately identifying 10 new molecules. Molecular docking was employed to explore the binding modes and targets between ligands and protein receptors, identifying interactions between amino acid residues forming hydrogen bond interactions and crystal structures. The newly designed compounds exhibited strong hydrogen bonding interactions with amino acid residues such as A/ALA213, A/VAL174, and A/LEU263, as well as hydrophobic interactions with residues like A/VAL147, A/LYS143, and A/LEU263, enhancing the binding strength between ligands and receptors. Molecular dynamics simulations were performed on four newly designed compounds, with results indicating that compound N3 bound to the 1MQ4 protein is the most stable. Snapshots were extracted from the lowest energy conformational state of the compounds in complex with protein 1MQ4. Finally, ADMET prediction was conducted for the 10 newly designed drug molecules, validating their high safety and pharmacological effects. This study provides insights for the design and development of novel Aurora kinase A inhibitor anticancer drugs, aiding in a better understanding of their inhibition mechanism, and offering a theoretical basis for synthesizing new anticancer drugs.

## Figures and Tables

**Table 1:** Compound structures and their activity values.

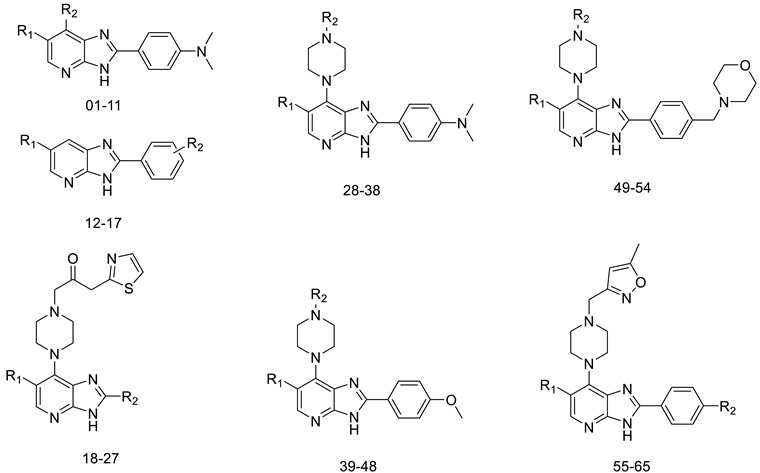			
NO.	R_1_	R_2_	Experimental p*IC*_50_
1	H	H	5.37
2 *	Cl	H	6.24
3	Br	H	6.31
4	CF3	H	6.13
5	Me	H	5.16
6 *	4-Methoxy-phenyl	H	4.70
7	Benzo[1,3]dioxole-5-yl	H	4.75
8	3-Hydroxy-phenyl	H	5.05
9	Cl	Cl	6.60
10 *	H	Me	5.16
11	H	Cl	5.62
12	H	m-Dimethylamino	5.00
13	H	p-Methoxy	5.18
14 *	H	m-Methoxy	4.80
15	H	o-Methoxy	4.54
16	H	p-Pyrrolidin-1-yl	5.34
17	H	p-Pyrid-2-yl	5.36
18 *	H	4-Dimethylamino-phenyl	6.06
19	Cl	4-Dimethylamino-phenyl	7.38
20	Br	4-Dimethylamino-phenyl	7.26
21	CN	4-Dimethylamino-phenyl	7.30
22 *	Cyclopropyl	4-Methoxy-phenyl	7.28
23	Br	H	6.29
24	Br	4-Dimethylaminomethyl-phenyl	7.80
25	Br	Ph	7.09
26 *	Cl	4-Methoxy-phenyl	7.28
27	Cl	4-Morpholin-4-ylmethyl-phenyl	8.52
28	Br	H	6.44
29	Cl	Phenylcarbamoylmethyl	6.94
30 *	Br	Pyridin-3-ylcarbamoylmethyl	6.93
31	Cl	(3-Chloro-phenylcarbamoyl)-methyl	7.13
32	Br	Phenylcarbamoyl	6.56
33	Br	Benzenesulfonyl	6.75
34 *	Br	Ph	6.59
35	Br	Isobutyl	6.68
36	Br	1-Pyridin-4-yl-ethyl	7.07
37	Cl	1-Phenyl-ethyl	7.10
38 *	Br	Pyridin-4-ylmethyl	7.26
39	Br	Pyridin-3-ylmethyl	8.05
40	Br	Pyrimidin-5-ylmethyl	8.10
41	Br	4-Chloro-benzyl	7.40
42 *	Br	Cyclopropylmethyl	6.19
43	Br	5-Methyl-isoxazol-3-ylmethyl	7.77
44	Br	2-Ethyl-oxazol-4-ylmethyl	7.09
45	Br	1-Methyl-4*H*-imidazol-2-ylmethyl	7.50
46 *	Br	Thiazol-4-ylmethyl	8.40
47	Cl	Pyridin-4-ylmethyl	7.68
48	Br	Pyridin-4-ylmethyl	7.82
49	Br	Pyridin-3-ylmethyl	8.30
50 *	Br	Pyrimidin-5-ylmethyl	8.22
51	Br	4-Chloro-benzyl	7.92
52	Br	5-Methyl-isoxazol-3-ylmethyl	8.70
53	Br	1-Methyl-4*H*-imidazol-2-ylmethyl	7.52
54 *	Br	Thiazol-4-ylmethyl	8.22
55	Br	Piperazin-1-ylmethyl	8.00
56	Br	Dimethylaminomethyl	7.85
57	Br	Aminomethyl	8.00
58 *	Cl	Aminomethyl	7.26
59	Br	2-Hydroxy-ethoxy	7.52
60	Cl	2-Hydroxy-ethoxy	7.54
61	Br	Piperazin-1-yl	8.00
62 *	Br	4-Methyl-piperazin-1-yl	7.82
63	Cl	4-Methyl-piperazin-1-yl	8.00
64	Br	Pyrazol-1-ylmethyl	8.52
65	Cl	Pyrazol-1-ylmethyl	8.00

* Test set.

**Table 2:** HQSAR model results using different fragment characteristic parameters for fixed holographic length (4–7).

Model	Fragment Distinction	Fragment Size	N	HL	*q* ^2^	*r* ^2^	SEE	SEEcv
1-01	A	4–7	6	83	0.87	0.957	0.247	0.432
1-02	B	4–7	6	151	0.812	0.933	0.310	0.519
1-03	C	4–7	5	307	0.870	0.964	0.226	0.427
1-04	A/B	4–7	4	71	0.844	0.937	0.293	0.463
1-05	A/C	4–7	6	257	0.861	0.967	0.217	0.446
1-06	A/H	4–7	2	401	0.800	0.844	0.453	0.512
**1-07**	**A/CH**	**4–7**	**6**	**151**	**0.872**	**0.961**	**0.237**	**0.428**
1-08	A/DA	4–7	4	199	0.859	0.948	0.267	0.440
1-09	CH/DA	4–7	5	199	0.860	0.955	0.251	0.443
1-10	A/B/C	4–7	5	257	0.861	0.960	0.237	0.442
1-11	A/B/H	4–7	2	401	0.796	0.839	0.459	0.517
1-12	A/B/CH	4–7	6	307	0.864	0.968	0.214	0.441
1-13	A/B/DA	4–7	3	61	0.841	0.926	0.314	0.462
1-14	A/C/H	4–7	2	53	0.794	0.832	0.470	0.519
1-15	A/C/CH	4–7	6	257	0.865	0.968	0.215	0.440
1-16	A/C/DA	4–7	3	199	0.850	0.921	0.325	0.448
1-17	A/H/CH	4–7	2	151	0.803	0.846	0.449	0.508
1-18	A/H/DA	4–7	3	151	0.832	0.907	0.353	0.474
1-19	A/B/C/H	4–7	4	97	0.805	0.900	0.371	0.516
1-20	A/B/C/CH	4–7	6	97	0.860	0.965	0.223	0.448
1-21	A/B/C/DA	4–7	3	71	0.839	0.916	0.335	0.464
1-22	A/B/H/CH	4–7	2	401	0.801	0.844	0.453	0.510
1-23	A/B/H/DA	4–7	3	59	0.833	0.894	0.377	0.473
1-24	A/B/CH/DA	4–7	3	151	0.839	0.922	0.324	0.465
1-25	A/C/CH/DA	4–7	2	199	0.849	0.898	0.366	0.445
1-26	A/H/CH/DA	4–7	3	151	0.835	0.904	0.358	0.470
1-27	A/B/C/H/CH	4–7	4	97	0.814	0.901	0.369	0.505
1-28	A/B/C/H/DA	4–7	5	97	0.863	0.946	0.276	0.438
1-29	A/B/C/CH/DA	4–7	6	59	0.863	0.962	0.235	0.440
1-30	A/B/H/CH/DA	4–7	3	401	0.828	0.901	0.365	0.480
1-31	A/C/H/CH/DA	4–7	3	199	0.834	0.902	0.362	0.472
1-32	A/B/C/H/CH/DA	4–7	5	97	0.832	0.941	0.287	0.485

**N:** Best composition score; **HL:** Holographic fragment length; ***q*^2^:** Cross-validation correlation coefficient; ***r*^2^:** Correlation coefficient of non-cross validation; **SEE:** Estimate standard error; **SEEcv:** Cross-validation standard error. Atom (A), chemical bond type (B), atom connection (C), hydrogen atom (H), chirality (CH), and as donor or acceptor (DA).

**Table 3:** HQSAR model analysis with the same fragment type (A/CH) and different fragment length.

Model	Fragment Distinction	Fragment Size	N	HL	*q* ^2^	*r* ^2^	SEE	SEEcv
1-07	A/CH	4–7	6	151	0.872	0.961	0.237	0.428
2-01	A/CH	1–3	6	83	0.772	0.882	0.412	0.572
2-02	A/CH	1–4	6	257	0.885	0.952	0.262	0.406
**2-03**	**A/CH**	**2–4**	**5**	**257**	**0.892**	**0.948**	**0.271**	**0.389**
2-04	A/CH	2–5	5	401	0.874	0.945	0.277	0.420
2-05	A/CH	3–6	6	307	0.884	0.959	0.243	0.409
2-06	A/CH	5–8	4	151	0.855	0.933	0.302	0.446
2-07	A/CH	6–9	6	199	0.881	0.967	0.219	0.414
2-08	A/CH	7–10	6	151	0.859	0.956	0.251	0.450
2-09	A/CH	8–11	6	353	0.868	0.966	0.222	0.436
2-10	A/CH	9–12	5	401	0.858	0.956	0.248	0.446

**Table 4:** Statistical Parameters of CoMFA and CoMSIA Models.

Model	Field	*q* ^2^	*r* ^2^	N	F	SEE	Contribution
CoMFA	S/E	0.866	0.983	6	403.587	0.156	0.486/0.514
3-01	S	0.847	0.922	4	130.757	0.326	1
3-02	E	0.804	0.965	6	193.892	0.224	1
3-03	H	0.729	0.931	5	116.254	0.311	1
3-04	D	0.198	0.982	8	2.454	0.982	1
3-05	A	0.490	0.728	3	40.052	0.604	1
3-06	S/E	0.864	0.965	5	234.646	0.223	0.372/0.628
3-07	S/H	0.833	0.953	4	222.533	0.254	0.487/0.513
3-08	S/D	0.841	0.939	5	132.909	0.292	0.828/0.172
3-09	S/A	0.846	0.939	5	131.767	0.293	0.535/0.465
3-10	E/H	0.831	0.982	6	380.156	0.161	0.610/0.390
3-11	E/D	0.819	0.977	6	301.906	0.18	0.840/0.160
3-12	E/A	0.815	0.952	5	169.461	0.26	0.672/0.328
3-13	H/D	0.722	0.974	8	190.455	0.196	0.838/0.162
3-14	H/A	0.810	0.986	8	361.979	0.143	0.538/0.462
3-15	D/A	0.482	0.774	3	51.450	0.55	0.222/0.778
3-16	S/E/H	0.867	0.977	5	364.504	0.18	0.263/0.458/0.279
3-17	S/E/D	0.855	0.974	5	319.939	0.192	0.321/0.545/0.134
3-18	S/E/A	0.859	0.970	6	227.267	0.207	0.276/0.453/0.271
3-19	S/H/D	0.833	0.985	9	283.698	0.153	0.424/0.450/0.127
3-20	S/H/A	0.852	0.966	5	241.583	0.22	0.332/0.321/0.346
3-21	S/D/A	0.863	0.958	7	132.490	0.25	0.489/0.125/0.386
3-22	E/H/D	0.832	0.994	8	857.566	0.093	0.538/0.341/0.122
3-23	E/H/A	0.839	0.982	6	381.917	0.161	0.443/0.290/0.266
3-24	H/D/A	0.839	0.989	8	466.395	0.126	0.476/0.104/0.421
3-25	S/E/H/D	0.860	0.986	5	589.859	0.142	0.236/0.398/0.256/0.110
3-26	S/E/H/A	0.867	0.984	6	427.260	0.152	0.208/0.346/0.231/0.215
3-27	S/E/D/A	0.871	0.985	9	283.928	0.152	0.241/0.419/0.098/0.242
3-28	S/H/D/A	0.865	0.991	8	525.498	0.119	0.286/0.311/0.112/0.291
3-29	E/H/D/A	0.860	0.994	8	802.661	0.097	0.403/0.273/0.095/0.229
**3-30**	**S/E/H/D/A**	**0.877**	**0.995**	**9**	**802.161**	**0.091**	**0.183/0.316/0.221/0.089/0.192**

S, E, H, D and A represent space field, electrostatic field, hydrophobic field, hydrogen bond donor field and acceptor field, respectively.

**Table 5:** Results of the Topomer CoMFA model.

N	*q* ^2^	*r* ^2^	*q*^2^ Stderr	*r*^2^ Stderr	F	SEE
6	0.905	0.971	0.36	0.20	369.402	0.199

**N:** Best composition score; ***q*^2^:** Cross-validation coefficient; ***r*^2^:** Non-cross-validation coefficient; ***q*^2^ stderr:** Standard error of cross-validation coefficient; ***r*^2^ stderr:** Standard error of non-cross validation coefficient; **F:** Statistical value of F test; **SEE:** Estimate standard error.

**Table 6:** Comparison of predicted activity value and residual value of HQSAR, CoMFA, CoMSIA and Topomer CoMFA models.

NO.	p*IC*_50_ Exp	HQSAR		COMFA		CoMSIA		Tomoper	
		p*IC*_50 Pred_	Residual	p*IC*_50 Pred_	Residual	p*IC*_50 Pred_	Residual	p*IC*_50 Pred_	Residual
01	5.37	5.14	0.23	5.54	−0.17	5.32	0.05	5.23	0.14
02 *	6.24	5.81	0.43	5.87	0.37	5.98	0.27	6.07	0.17
03	6.31	5.83	0.48	5.87	0.44	6.12	0.19	6.03	0.28
04 *	6.13	6.02	0.11	6.27	−0.14	6.31	−0.18	6.02	0.11
05	5.16	5.45	−0.29	5.56	−0.40	5.35	−0.19	5.55	−0.39
06 *	4.70	5.05	−0.35	5.15	−0.45	5.24	−0.54	4.86	−0.16
07	4.75	4.77	−0.03	4.64	0.11	4.74	0.01	4.74	0.00
08 *	5.05	4.98	0.07	4.92	0.12	5.03	0.02	4.98	0.07
09	6.60	6.42	0.18	6.18	0.42	6.43	0.17	6.55	0.05
10 *	5.16	5.34	−0.19	5.72	−0.56	5.41	−0.26	5.39	−0.23
11	5.62	5.92	−0.30	5.85	−0.23	5.78	−0.16	5.68	−0.06
12	5.00	5.14	−0.14	4.97	0.03	4.98	0.02	4.82	0.18
13	5.18	4.96	0.22	5.18	0.00	5.11	0.07	5.44	−0.26
14 *	4.80	4.96	−0.16	5.02	−0.22	4.82	−0.02	4.80	0.00
15	4.54	4.89	−0.35	4.60	−0.06	4.53	0.01	4.53	0.01
16	5.34	5.58	−0.24	5.47	−0.13	5.40	−0.06	5.49	−0.15
17	5.36	5.37	−0.01	5.32	0.04	5.29	0.06	5.41	−0.05
18 *	6.06	6.66	−0.60	6.54	−0.48	6.52	−0.46	6.53	−0.47
19	7.38	7.43	−0.05	7.27	0.11	7.28	0.09	7.40	−0.02
20	7.26	7.49	−0.23	7.30	−0.04	7.45	−0.19	7.36	−0.10
21	7.30	7.54	−0.24	7.31	−0.01	7.26	0.04	7.61	−0.31
22 *	7.28	6.82	0.46	6.89	0.39	6.26	1.02	6.96	0.32
23	6.29	6.52	−0.23	6.28	0.01	6.30	−0.01	6.46	−0.17
24	7.80	7.76	0.04	7.82	−0.03	7.81	−0.01	7.71	0.09
25	7.09	7.24	−0.14	6.98	0.11	7.12	−0.03	6.96	0.13
26 *	7.28	7.25	0.03	7.27	0.01	7.16	0.12	7.61	−0.33
27	8.52	8.04	0.49	8.55	−0.03	8.49	0.04	8.25	0.27
28	6.44	6.64	−0.21	6.58	−0.14	6.43	0.01	6.60	−0.16
29	6.94	6.69	0.25	6.83	0.11	6.94	0.00	6.74	0.20
30 *	6.93	7.30	−0.37	7.01	−0.08	7.41	−0.48	7.21	−0.28
31	7.13	7.41	−0.29	7.09	0.04	7.16	−0.03	7.22	−0.09
32	6.56	6.92	−0.36	6.45	0.11	6.55	0.00	6.47	0.09
33	6.75	6.92	−0.17	6.76	−0.01	6.77	−0.02	6.61	0.14
34 *	6.59	6.75	−0.16	6.84	−0.25	6.84	−0.25	6.80	−0.21
35	6.68	6.45	0.23	6.76	−0.08	6.72	−0.04	6.72	−0.04
36	7.07	6.93	0.14	6.97	0.11	7.04	0.03	6.90	0.17
37	7.10	6.61	0.49	7.14	−0.04	7.10	0.00	7.30	−0.20
38 *	7.26	7.61	−0.35	7.72	−0.46	7.81	−0.55	7.41	−0.15
39	8.05	7.00	1.05	8.03	0.01	7.98	0.06	7.87	0.18
40	8.10	8.04	0.06	8.13	−0.04	8.14	−0.04	7.88	0.22
41	7.40	7.53	−0.13	7.53	−0.13	7.39	0.01	7.50	−0.10
42 *	6.19	7.16	−0.98	7.13	−0.94	7.33	−1.14	7.08	−0.89
43	7.77	7.54	0.23	7.74	0.03	7.78	−0.01	7.75	0.02
44	7.09	7.32	−0.23	7.16	−0.07	7.08	0.02	7.47	−0.38
45	7.50	7.09	0.40	7.60	−0.11	7.48	0.02	7.13	0.37
46 *	8.40	7.27	1.13	7.45	0.95	7.59	0.81	7.29	1.11
47	7.68	7.37	0.31	7.70	−0.03	7.64	0.04	7.65	0.03
48	7.82	7.43	0.39	7.72	0.11	7.79	0.04	7.61	0.21
49	8.30	8.49	−0.19	8.41	−0.11	8.24	0.06	8.51	−0.21
50 *	8.22	8.82	−0.60	8.50	−0.28	8.38	−0.16	8.52	−0.30
51	7.92	8.31	−0.39	7.94	−0.02	7.91	0.02	8.14	−0.22
52 *	8.70	8.32	0.38	8.64	0.06	8.74	−0.04	8.40	0.30
53	7.52	7.88	−0.35	7.63	−0.10	7.49	0.03	7.77	−0.25
54 *	8.22	8.32	−0.10	7.83	0.39	8.23	−0.01	7.94	0.28
55	8.00	8.05	−0.05	8.04	−0.04	8.02	−0.02	7.99	0.01
56	7.85	7.98	−0.13	7.89	−0.04	7.87	−0.02	7.89	−0.04
57	8.00	7.90	0.10	7.87	0.13	7.99	0.01	7.71	0.29
58 *	7.26	7.62	−0.36	7.77	−0.51	7.62	−0.36	7.74	−0.48
59	7.52	7.72	−0.20	7.60	−0.07	7.57	−0.04	7.55	−0.03
60	7.54	7.66	−0.12	7.58	−0.04	7.44	0.09	7.59	−0.05
61	8.00	8.05	−0.05	8.02	−0.02	8.02	−0.02	7.99	0.01
62 *	7.82	8.08	−0.25	8.04	−0.22	8.15	−0.33	7.98	−0.16
63	8.00	8.02	−0.02	8.03	−0.03	8.02	−0.02	8.02	−0.02
64	8.52	8.27	0.25	8.14	0.38	8.39	0.14	8.37	0.15
65	8.00	8.21	−0.21	8.12	−0.12	8.23	−0.23	8.41	−0.41

**Table 7:** External verification of the QSAR Model.

Parameters	Criterion	HQSAR	COMFA	CoMSIA	Topomer
$r_{pred}^{2}$	$r_{pred}^{2}>0.6$	0.814	0.829	0.758	0.855
*k*	0.85 < *k* < 1.15	0.977	0.980	0.978	0.984
*k*′	0.85 < *k*′ < 1.15	1.018	1.015	1.017	1.012
$r_{0}^{2}$	$\frac{\left(r^{2}\;-\;r_{0}^{2}\right)}{r^{2}}<0.1$	0.828	0.838	0.803	0.860
$r_{0}^{2^{\prime }}$	$\frac{\left(r^{2}\;-\;r_{0}^{2^{\prime }}\right)}{r^{2}}<0.1$	0.807	0.779	0.770	0.836
$r_{m}^{2}$	$r_{m}^{2}>0.5$	0.610	0.609	0.559	0.647
$r_{m}^{2^{\prime }}$	$r_{m}^{2^{\prime }}>0.5$	0.584	0.539	0.523	0.615
${∆r}_{m}^{2}$	${∆r}_{m}^{2}<0.2$	0.026	0.070	0.036	0.033
RMSE	RMSE → 0	0.501	0.480	0.530	0.443
MAE	MAE → 0	0.543	0.547	0.565	0.462
CCC	CCC > 0.85	0.902	0.902	0.888	0.922

**Table 8:** Molecular structure of the new compounds and activity value prediction using the Topomer CoMFA model.

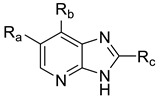							
Molecule	R_a_	R_b_	R_c_	Contribution Value			p*IC*_50 pred_
				R_a_	R_b_	R_c_	
N1	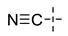	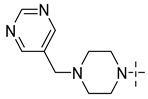	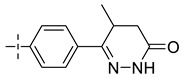	0.80	1.54	2.20	9.27
N2	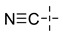	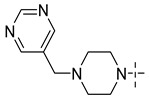	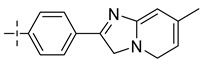	0.80	1.54	2.14	9.23
N3	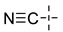	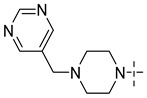	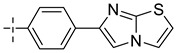	0.80	1.54	2.10	9.18
N4	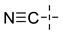	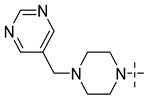	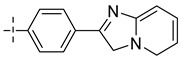	0.80	1.54	2.05	9.14
N5	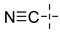	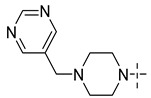	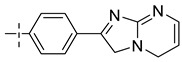	0.80	1.54	2.02	9.11
N6	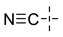	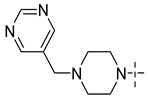	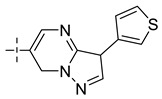	0.80	1.54	1.97	9.04
N7	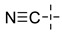	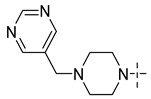	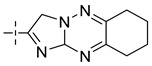	0.80	1.54	1.95	9.02
N8	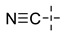	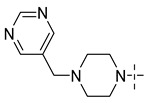	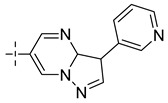	0.80	1.54	1.93	8.98
N9	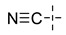	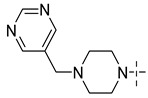	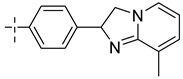	0.80	1.54	1.92	9.00
N10	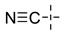	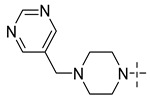	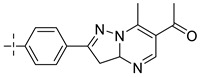	0.80	1.54	1.91	8.96

**Table 9:** Molecular docking results and scoring function.

Molecule	p*IC*_50 Pred_	Contribution Value			Scoring Function		
		R_a_	R_b_	R_c_	Total-Score	Crash	Polar
Template	8.70	0.80	1.54	1.77	6.3210	−1.7537	1.2747
N1	9.27	1.08	1.68	2.20	8.1293	−2.0803	0.5836
N2	9.23	1.08	1.68	2.14	5.4437	−1.6456	1.1618
N3	9.18	1.08	1.68	2.10	7.8842	−1.4843	2.2739
N4	9.14	1.08	1.68	2.05	7.2484	−2.5719	2.4922
N5	9.11	1.08	1.68	2.02	7.1447	−1.5927	1.1425
N6	9.04	1.08	1.68	1.95	4.6770	−2.6022	1.1778
N7	9.02	1.08	1.68	1.93	7.5526	−2.0592	2.3005
N8	8.98	1.08	1.68	1.92	5.8277	−3.2544	1.9236
N9	9.00	1.08	1.68	1.90	5.8477	−3.1327	1.4617
N10	8.96	1.08	1.68	1.89	6.4953	−1.6457	1.9826

**Total-score:** Rank of the affinity of ligands that bind to the active site of the receptor and report the output of the total score. **Crash:** Crash score shows inappropriate penetration into the binding site. A crash score close to 0 is advantageous. A negative number means penetration. **Polar:** Polarity indicates the contribution of polar interactions to the total score.

**Table 10:** The binding energies for newly designed compounds (N3, N4, N5, N7) combined with PCAF protein (5TPX) by MM/PBSA study.

Energy Terms (kcal/mol)	52	N3	N4	N5	N7
${∆E}_{vdw}$	−50.813 ± 4.865	−172.076 ± 1.610	−193.759 ± 1.693	−191.519 ± 1.474	−174.278 ± 1.446
${∆E}_{elec}$	−61.006 ± 2.977	−56.241 ± 4.117	−47.689 ± 1.434	−86.251 ± 3.671	−52.961 ± 1.484
${∆E}_{polar}$	90.704 ± 6.468	178.769 ± −4.801	208.975 ± 2.246	237.124 ± 4.049	195.046 ± 5.573
${∆E}_{SASA}$	−5.824 ± 0.532	−18.960 ± 0.157	−22.428 ± 0.122	−21.198 ± 0.172	−20.861 ± 0.172
$∆G_{bind}$	−26.939 ± 5.302	−68.515 ± 2.218	−54.869 ± 1.705	−62.317 ± 2.418	−52.857 ± 4.201

**Table 11:** ADMET prediction results.

Molecular	Absorption		Distribution		Metabolism			Excretion		Toxicity	
	HIA	F	PPB	BBB	CYP450 3A4	CYP450 2D6	CYP450 2C9	T1/2	CL	AMES	Carcinogenicity
N1	99.43%	58.57%	0.975	0.986	Inhibitor	Non	Inhibitor	0.784	6.378	Non	Non
N2	98.61%	58.57%	0.955	0.822	Inhibitor	Non	Inhibitor	0.733	9.318	Non	Non
N3	96.67%	61.43%	0.903	0.996	Inhibitor	Non	Inhibitor	0.489	8.638	Non	Non
N4	95.80%	50.00%	1.001	0.992	Inhibitor	Non	Inhibitor	0.709	8.848	Non	Non
N5	96.75%	55.71%	0.997	0.991	Inhibitor	Non	Inhibitor	0.442	7.315	Non	Non
N6	92.07%	58.57%	1.042	0.989	Inhibitor	Non	Inhibitor	0.408	6.781	Non	Non
N7	97.73%	57.14%	0.817	0.994	Inhibitor	Non	Inhibitor	0.249	3.946	Non	Non
N8	93.23%	55.71%	0.883	0.990	Inhibitor	Non	Inhibitor	0.594	4.794	Non	Non
N9	98.91%	58.57%	0.866	1.000	Inhibitor	Non	Inhibitor	0.158	6.421	Non	Non
N10	92.65%	58.57%	0.929	0.988	Inhibitor	Non	Inhibitor	0.301	5.548	Non	Non

**HIA**: ≥30% (well absorbed); **F**: ≥30% (high bioavailability); **PPB:** ≥90 (highly integrated); **BBB:** ≥0.1 (high ratio); **T1/2:** >0.5 (suggestions); **CL:** >15 mL/min/kg: high, 5 mL/min/kg < moderate < 15 mL/min/kg, <5 mL/min/kg: low.

## Data Availability

All data generated or analyzed during this study are included in the article.

## Supplementary Materials

The following supporting information can be downloaded at: https://www.mdpi.com/article/10.3390/molecules29081772/s1, Figure S1: 2D interaction diagram of compounds N3, N4, N5, and N7 docked with receptor proteins; Table S1: The mean and standard deviation values of RMSD and Rg of every Complex.
