# Supplementary material for: QSAR Study, Molecular Docking and Molecular Dynamic Simulation of Aurora Kinase Inhibitors Derived from Imidazo[4,5-b]pyridine Derivatives
Source: Molecules. 2024 Apr 13;29(8):1772. doi: 10.3390/molecules29081772 (PMC11052498; doi:10.3390/molecules29081772)
Supplement: Supplementary file 1 [file molecules-29-01772-s001.zip › molecules-2931956-supplementary.pdf]

Table S1 The mean and standard deviation values of RMSD and Rg of every Complex

	RMSD		Rg	
	Standard deviation value	Average value	Standard deviation value	Average value
52	0.457	1.595	0.0909	0.895
N3	0.0492	0.233	0.0870	1.043
N4	0.0567	0.378	0.0833	1.049
N5	0.0691	0.276	0.0657	1.187
N7	0.0567	0.378	0.0662	0.974

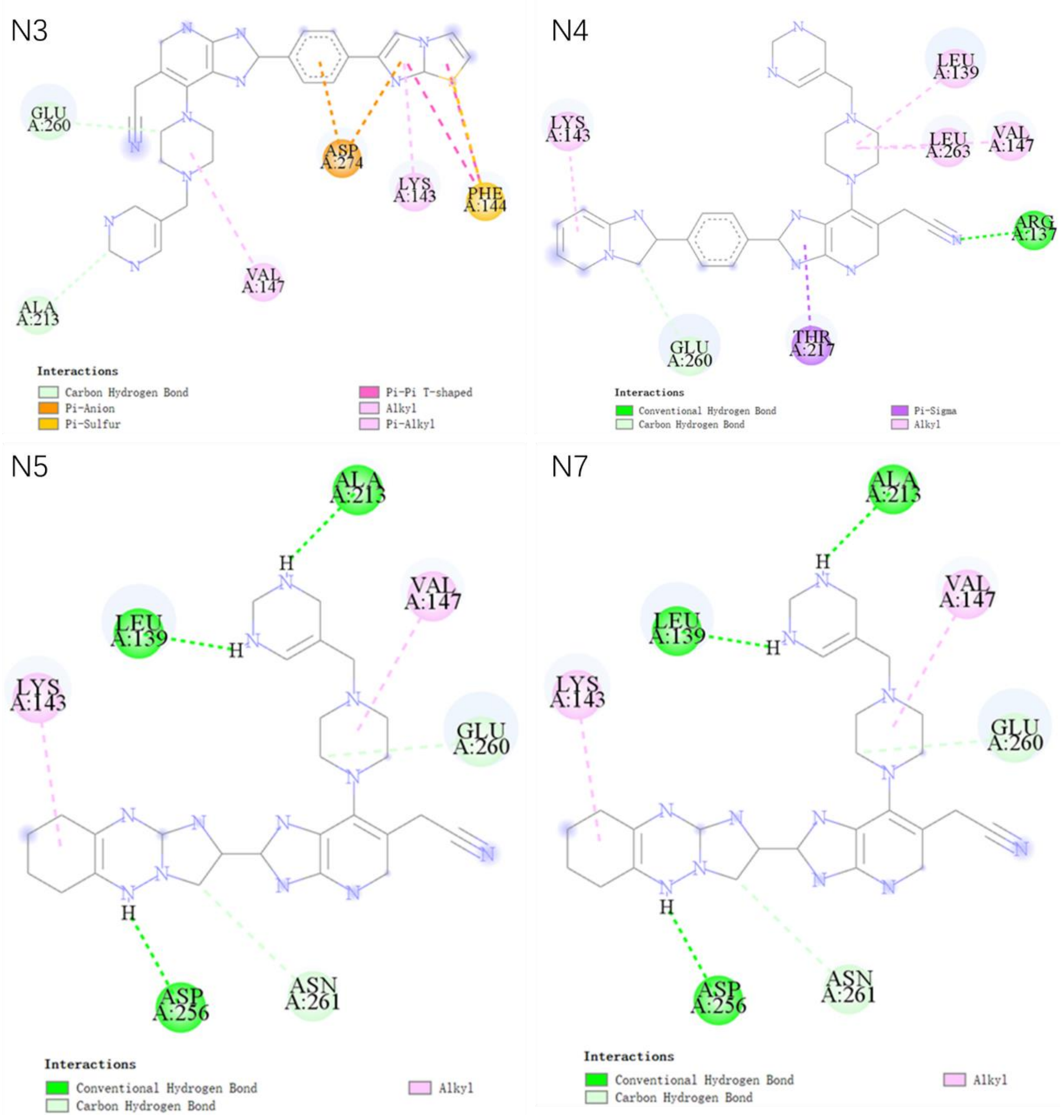

Figure S1. 2D interaction diagram of compounds N3, N4, N5, and N7 docked with receptor proteins
